# Therapeutic Notes

**Published:** 1895-04-13

**Authors:** 


### THERAPEUTIC NOTES.

### PAPAIN.

Papain is a ferment closely resembling pepsin, obtained from the juice of the papaw tree (*Carica papaya*). It is a yellowish-white powder, having a somewhat aromatic taste, and is given in doses of two to eight grains. Sittmann (Munch. Med. Wochenschr., 1893) has made numerous observations on its digestive power and therapeutical value. It acts in a faintly alkaline or in an acid medium, and it was found that two-thirds of a grain was sufficient to convert 150 grains of coagulated white of egg into a milky fluid in two hours, no unchanged albumin remaining. Given in four to eight grain doses immediately after meals, it was used in cases of acute gastric catarrh with excellent results, the relief being almost immediate, and cure occurring in two or three days. Chronic cases were more obdurate, but generally yielded to treatment within a fortnight. Two cases of cancer were relieved as regards symptoms, but in dilatation of the stomach, when combined with electricity and massage the curative results were permanent. In gastric neurosis little benefit was obtained. It is difficult to gather what part diet played in the treatment of some of the cases, and, therefore, in the results.

